# Higher Levels of Multiple Paternities Increase Seedling Survival in the Long-Lived Tree *Eucalyptus gracilis*

**DOI:** 10.1371/journal.pone.0090478

**Published:** 2014-02-28

**Authors:** Martin F. Breed, Matthew J. Christmas, Andrew J. Lowe

**Affiliations:** 1 Australian Centre for Evolutionary Biology and Biodiversity (ACEBB) and School of Earth and Environmental Sciences, University of Adelaide, Adelaide, South Australia, Australia; 2 State Herbarium of South Australia, Science Resource Centre, Department of Environment, Water and Natural Resources, Adelaide, South Australia, Australia; CNR, Italy

## Abstract

Studying associations between mating system parameters and fitness in natural populations of trees advances our understanding of how local environments affect seed quality, and thereby helps to predict when inbreeding or multiple paternities should impact on fitness. Indeed, for species that demonstrate inbreeding avoidance, multiple paternities (*i.e.* the number of male parents per half-sib family) should still vary and regulate fitness more than inbreeding – named here as the ‘constrained inbreeding hypothesis’. We test this hypothesis in *Eucalyptus gracilis*, a predominantly insect-pollinated tree. Fifty-eight open-pollinated progeny arrays were collected from trees in three populations. Progeny were planted in a reciprocal transplant trial. Fitness was measured by family establishment rates. We genotyped all trees and their progeny at eight microsatellite loci. Planting site had a strong effect on fitness, but seed provenance and seed provenance × planting site did not. Populations had comparable mating system parameters and were generally outcrossed, experienced low biparental inbreeding and high levels of multiple paternity. As predicted, seed families that had more multiple paternities also had higher fitness, and no fitness-inbreeding correlations were detected. Demonstrating that fitness was most affected by multiple paternities rather than inbreeding, we provide evidence supporting the constrained inbreeding hypothesis; *i.e.* that multiple paternity may impact on fitness over and above that of inbreeding, particularly for preferentially outcrossing trees at life stages beyond seed development.

## Introduction

The realised inbreeding rate of monoecious trees, when estimated from mature seeds or seedlings, is usually constrained below their actual inbreeding rate [1]. This occurs because inbreeding usually imposes fitness costs at early stages of reproduction [1]–[4], via expression of deleterious recessive alleles [5]–[7], leading to the abortion of these inbred offspring. Selfing and biparental inbreeding (*i.e.* related breeding events) should both be constrained, but selfing more so than biparental inbreeding due to the higher inbreeding coefficients that are generated during selfing.

Monoecious trees also routinely exhibit multiple paternities because they receive great amounts of pollen from a large diversity of donors [8]–[10]. Regulation of the supply and diversity of pollen is largely controlled by a tree’s local environment, often regulated by local variation in pollination services and the effective density of pollen donors [11], [12]. Fertilisation success is then filtered by the availability of receptive flowers (*i.e*. tree phenology) and the genetic compatibility of pollen-ovule combinations.

Tree fitness should increase with more multiple paternities because, firstly, higher levels of multiple paternities should facilitate more complimentary pollen-ovule combinations by generating greater opportunities for female choice for superior pollen and/or pollen competition [8], [13]. Thus, females that have lower levels of multiple paternities have, by definition, placed greater weight on their compatibility with fewer pollen donors and undergone suboptimal levels of mate discrimination – the bet hedging hypothesis [13]. Secondly, higher levels of multiple paternities should also give rise to greater genetic diversity within progeny arrays (*i.e.* greater genotype × environment interactions within progeny arrays) [13]. Theory predicts that greater offspring genetic diversity should facilitate higher mean offspring fitness as a result of, for example, more effective resource exploitation from offspring [13]. Consequently, females that receive lower levels of multiple paternities should have less genetically diverse offspring, increasing the risk that a high proportion of her offspring will be poorly adapted to local environments, particularly in unpredictable or changing environments.

Given that multiple paternities are likely to impose fitness benefits, and that trees usually maintain low realised inbreeding levels, divergence from this high multiple paternity state is expected to produce lower fitness offspring - named here as the ‘constrained inbreeding hypothesis’. Furthermore, trees should be good candidates to detect the effect of multiple paternities because trees tend to have large lifetime fecundities, which result in strong selection acting at early life stage (*e.g.* seedlings) [9], [14].

Inbreeding and inbreeding depression are possibly present at late stages of offspring development in natural tree populations (*e.g.* [15]), but there has been little emphasis on the potential magnitude of fitness effects of multiple paternities in natural tree populations. Indeed, there are numerous examples of fragmented tree populations where mature seed/seedling fitness was studied (*i.e*. after the effects of early inbreeding depression), but the studied trees maintained strong inbreeding avoidance [11], [16], [17]. Consequently, there was insufficient variation in inbreeding to observe inbreeding-fitness correlations in these studies [18], but there were detectable effects of multiple paternities on fitness [11], [16], [17]. These are significant observations since seeds and seedlings are both important life stages for the demographic trajectories of tree populations [19], and both are critical life stages for land managers who require good quality trees (*e.g*. for ecological restoration; [20], [21]).

Variation in local pollination services can greatly affect the extent of multiple paternities, related breeding events, and selfing observed in trees [22]–[25]. Furthermore, over 20 years of effort has been invested into reporting changes in local pollination by estimating tree mating system parameters in fragmented populations [11], [26]–[28].

Despite the established theory of optimal pollinator foraging behaviour in natural populations [29], [30], as well as the observed fitness impacts due to shifts in pollinator foraging in fragmented populations [11], [16], [17], [22], [31]–[34], it is surprising that not more studies have explored how natural variation in these mating system parameters may impact on fitness of intact tree populations. Indeed, it may be that natural populations of trees also tend to experience minimal inbreeding depression at later life stages (*i.e.* after seed development) because of strong early inbreeding depression [1]–[4], but this is currently unknown. As a consequence of this expectation, the degree of multiple paternities may have stronger effects on fitness at late life stages than inbreeding (*e.g.* mature seed, seedling), even in natural populations of trees.

**Figure 1:**
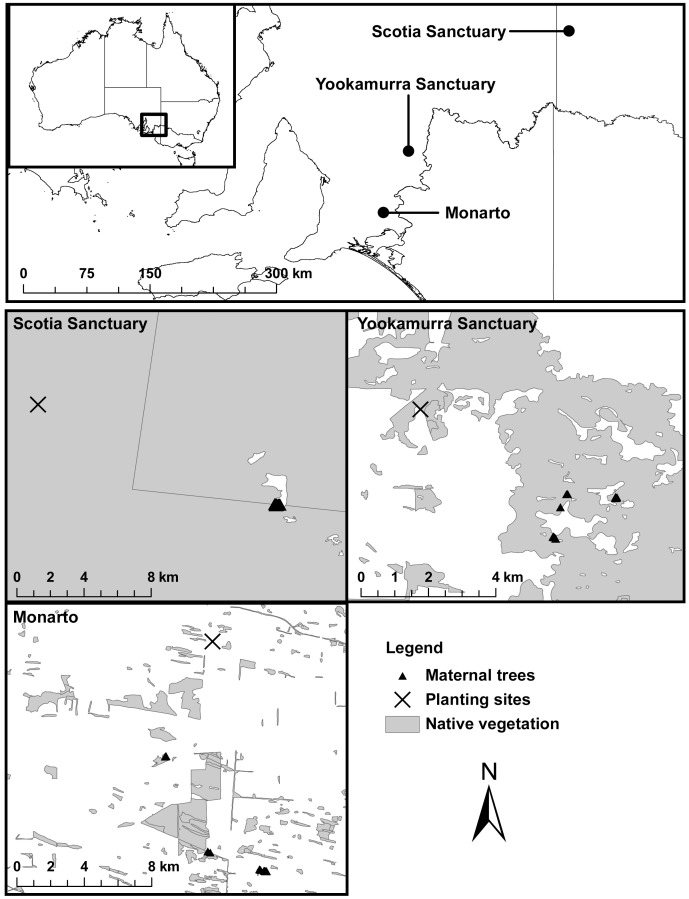
Map showing location of *Eucalyptus gracilis* maternal trees and planting sites. Maps show samples from the three populations in the Murray Darling Basin, Australia. Insert maps show greater spatial information on sampled populations. Reciprocal transplant planting locations shown at each planting site by a cross (x).

To test whether multiple paternities have stronger effects on fitness at late life stages than inbreeding, we combined assessments of mating system parameters and seedling establishment rates of open-pollinated progeny arrays (measured 16 months after germination) of *Eucalyptus gracilis* F. Muell. (white mallee or yorrell). We estimated four different mating system parameters; two commonly used measures of multiple paternities (correlated paternity and the number of full-sibships) and two measures of inbreeding (outcrossing rate and biparental inbreeding). We sampled progeny arrays from three natural and mostly intact populations across the Murray-Darling Basin in southern Australia (Figure 1). Like other eucalypts [3], [25], we expect that *E. gracilis* will express strong inbreeding avoidance, constraining variation in the measured inbreeding parameters, and therefore we expect to detect high levels of outcrossing and low levels of biparental inbreeding. This inbreeding constraint should reduce the probability of detecting inbreeding-seedling fitness correlations [18]. However, the levels of multiple paternities within progeny arrays of *E. gracilis* should not be constrained, but rather should vary across the families we sampled according to their local pollination services. Additionally, *E. gracilis* should be a good candidate to explore the effects of inbreeding and multiple paternities because, like other eucalypts, *E. gracilis* should be a strong outcrosser and have high lifetime fecundity [3], [35], and as a consequence of these life history traits, *E. gracilis* offspring should have significant genetic load [5] and strong selection should act at the seedling life stage [9]. We therefore expect that this study system is suitable for isolating and detecting multiple paternity-seedling fitness correlations.

## Materials and Methods

### Study Species

*Eucalyptus gracilis* is a multi-stemmed, sclerophyllous tree common throughout the sand and sand-over-limestone soils of the Murray-Darling Basin, southern Australia [36], [37]. *Eucalyptus gracilis* generally grows from 4 to 8 m high, it has small white hermaphroditic flowers (diameter of mature flowers with reflexed stamens: <15 mm) and is pollinated primarily by small insects and, to a lesser degree, by birds and small marsupials [38], [39].

Eucalypt flowers are protandrous (*i.e.* male reproductive phase precedes female phase within flowers) and flower development within and between inflorescences is sequential and gradual. Therefore, flowers in male or female phase may be in close proximity, allowing geitonogamous selfing to occur (*i.e.* pollination from another flower on the same plant; [40]). Data from closely related eucalypts suggest that the species investigated here probably has a late-acting self-incompatibility mechanism, resulting in mixed mating to preferential outcrossing (*t*_m_ generally >0.70; [3]). Serotinous fruit (*i.e.* seeds released in response to an environmental trigger) are held over numerous years, with drying triggering seed-release. Seeds are small (<2 mm diameter) and gravity dispersed. Based on data from *E. incrassata* and our field observations, ants generally exhaust soil seed banks, except during particularly heavy seed release such as post fire [41], [42].

### Seed Collection

Open-pollinated seeds were collected from across the canopies of trees located in three sites in the Murray-Darling Basin (Figure 1). Scotia Sanctuary and Yookamurra Sanctuary trees (*n _Scotia_* = 18; *n _Yookamurra_* = 20; Figure 1) were from large intact woodlands, with no history of known anthropogenic disturbance. Monarto Woodland trees (*n* = 20; Figure 1) were from small remnant woodlands. Small remnant woodlands were natural habitats surrounded by agricultural land, but again with no history of known anthropogenic disturbance. *E. gracilis* is a common overstory tree at each site (N >1000), and one of many *Eucalyptus* species common throughout the semi-arid Murray Darling Basin [36]. We avoided sampling nearest neighbours and we sampled numerous stands per site where possible, although road access limited our sampling to one stand at Scotia Sanctuary. *E. gracilis* stands at Yookamurra Sanctuary had significant higher density than stands at both Scotia Sanctuary and Monarto Woodland (trees ha^−1^: Monarto Woodland = 23.67, SD = 2.29; Yookamurra Sanctuary = 49.33, SD = 4.63; Scotia Sanctuary = 20.42, SD = 3.24).

### Seedling Establishment Trials

Fifteen replicates of approximately 20 seeds from each tree were sown on February 1^st^ 2010. Germination was conducted under semi-controlled glasshouse conditions in Adelaide, South Australia (S34°55’05″, E138°36’18″). All seedlings were moved to full-sun at the Mt Lofty Botanic Gardens, South Australia (S34°59’03″, E138°43’08″) after four weeks in glasshouse conditions. Crates of seedlings were shifted and rotated approximately weekly to avoid confounding effects of location in glasshouse/nursery. The most central seedling within each pot was chosen, and non-central additional seedlings were removed over the subsequent weeks prior to planting. We hoped to minimise selection on seedling fitness with this process, but cannot rule out that selection for fitter individuals may have taken place. Glasshouse and nursery environments may allow inferior seedlings to survive when compared to seedling survival under natural woodland conditions. This bias should be consistent across progeny arrays and, under glasshouse/nursery environments, additional biases should be controlled for (*e.g*. competition, demographic or environmental stochastic effects).

Plantings took place at Scotia Sanctuary, Yookamurra Sanctuary and at Monarto Woodland between May and June 2010 (seed source sample sizes: *n*_Monarto Woodland_ = 294; *n*_Yookamurra_ = 295; *n*_Scotia = _264; reciprocal transplant experiment locations shown in Figure 1). We implemented a randomised complete block design [43]. Planting sites were located in close proximity to ‘local’ maternal trees (<13 km in all cases; Figure 1). Planting sites were prepared by rotary hoeing to remove residual surface vegetation, parallel rip-lines were drawn through at 3 m intervals, and seedlings were spaced at 2 m intervals. A 200×200×500 mm tree guard (Global Land Repairs, Fyshwick) surrounded each seedling to protect against herbivores (*e.g*. rabbits). This reciprocal transplant experiment was originally planned to explore adaptive divergence in *E. gracilis* as well as this mating system analysis. However, since we found such weak neutral genetic differentiation and no divergence in establishment rate (see results presented below), this investigation was set aside and the mating system analysis was conducted in more depth.

In May 2011, we counted the number of seedlings that had died 12 months after planting (*i.e.* 16 months after germination). This fitness proxy included deaths that had occurred at each planting site, whether local or non-local. We used the ratio of mortality counts and progeny array size as the variable in subsequent analyses. Using seedling mortality as our only fitness proxy means that we can only speculate about earlier or later stages of the life cycle of *E. gracilis* (*e.g.* germination, fecundity), and therefore our results need to be interpreted in this context.

### Microsatellite Genotyping

Leaf tissue was collected from each seedling prior to planting and DNA was extracted using the Machery-Nagel Nucleospin Plant II Kit at the Australian Genome Research Facility (AGRF, Adelaide, Australia). Eight direct-labelled microsatellite markers were selected from the set of EST-derived markers by Faria *et al.* ([44]; EMBRA1382; EMBRA2002; EMBRA1445; EMBRA1284; EMBRA1928; EMBRA1468; EMBRA1363). A BLAST search was performed for each microsatellite sequence using accession numbers in Faria *et al.* [44] and resulted in no significant hits with genes with a known function. EMBRA1363 produced two unlinked and scoreable PCR products (EMBRA1363a and b). PCR was performed in a single 10 µL multiplex PCR containing 1 µL template DNA (*ca.* 20 ng µL^−1^), 5 µL 2× Qiagen Multiplex PCR Master Mix (Qiagen, Hilden, Germany), 3 µL of nuclease-free water, 1 µL of primer mix with each primer at 2 µM concentration. Standard Qiagen Multiplex PCR conditions were used with an initial activation step at 95°C for 15 minutes, 40 cycles of denaturation at 94°C for 30 seconds, annealing at 60°C for 90 seconds and extension at 60°C for 60 seconds, with final extension at 60°C for 30 minutes. LIZ500 size standard was added to samples and fragments were separated on an AB3730 genetic analyser with a 36 cm capillary array (Applied Biosystems, Foster City, MA, USA) at AGRF. Alleles were automatically called using GeneMapper software (Applied Biosystems) and double-checked manually.

### Data Analysis

Each maternal tree was presumed to reflect patterns of population genetics pre-clearance since all sampled trees were estimated to be >80 years old [45], [46] and most land clearance occurred <80 years ago [47]. Maternal genotypes were used to screen for null alleles in MICRO-CHECKER [48] and INEst [49], where INEst employs a method that produces un-biased estimates of null allele frequencies for populations that experience inbreeding. GENEPOP on the web (http://genepop.curtin.edu.au) was used for tests for heterozygote deficit/excess and linkage disequilibrium, applying sequential Bonferroni correction for multiple testing where appropriate. Additionally, the per-locus probability of paternity exclusion (*Q*) and combined probability of paternity exclusion (*QC*) were estimated in GENALEX [50]. Pairwise population genetic differentiation parameters *G_ST_est_* [51] and *D*_est_ [52] were estimated in GENODIVE [53].

We estimated the following genetic diversity parameters for maternal tree and progeny groups using GENALEX: number of alleles (*A*), Nei’s unbiased expected and observed heterozygosity (*H*_E_ and *H*_O_, respectively; [54]). In addition, the fixation index (*F*) was estimated for each population. To account for differences in sample size, we estimated the rarefied mean number of alleles per locus (*AR*) using HP-RARE [55]. All samples that failed amplification at more than three loci were excluded (*n* = 5).

We estimated the following mating system parameters in MLTR [56]: multilocus outcrossing rate (*t*_m_), biparental inbreeding (*t_m_–t_s_*) and multilocus correlated paternity (*r*_p_). Families were bootstrapped 1000 times to calculate variance estimates for each parameter. Family-level mating system parameters were estimated in the same way except that individuals within families were bootstrapped 1000 times to calculate variance estimates. To further investigate the role of the multiple paternities, we estimated the number of full-sib groups within progeny arrays using KINALYZER [57], [58], implementing the 2-allele algorithm, and scaled this value to the size of progeny arrays (*k_n_*). Selfed offspring were excluded from this analysis.

We used general linear models in a maximum likelihood, multi-model inference framework in R v. 2.12.1 (R Project for Statistical Computing, http://www.r-project.org; [59] to test for hypothesised relationships between *E. gracilis* establishment success (counts of seedling mortality per family, scaled to size of family) and four genetic predictors: multilocus outcrossing rate (*t*_m_), biparental inbreeding (*t_m_–t_s_*), correlated paternity (*r*_p_) and the number of full-sibships within progeny arrays scaled to size of progeny array (*k_n_*). We relied on Akaike’s Information Criterion corrected for small sample sizes (AIC*_c_*) for model selection [59].

### Ethics Statement

All relevant permits and approvals were obtained for the work presented in this study. Work conducted in Scotia and Yookamurra Sanctuary was done with written approval from the landowner, Australian Wildlife Sanctuary. Work conducted in Monarto Woodland was approved by Primary Industries and Regions SA (PIRSA-ForestrySA and Rural Solutions). Work conducted on Ferries-McDonald Conservation Park and Monarto Conservation Park was approved by the South Australian Department of Environment and Heritage (now Department of Environment, Water and Natural Resources). No protected species were sampled.

### Data Access

Data accession numbers have not yet been obtained, but they will be provided in the event that our manuscript is accepted for publication.

## Results

### Genetic Marker Quality

We genotyped open-pollinated progeny from 20 trees from Monarto Woodland (*n* = 287), 20 trees Yookamurra Sanctuary (*n* = 291) and 18 trees from Scotia Sanctuary (*n* = 260) (progeny array size data reported in Table 1). A total of 115 different alleles were identified across progeny (Table S1). The combined probability of paternity exclusion if neither parent is known indicates good resolution for the genetic markers used (QC = 1.00). No significant excesses or deficits of heterozygotes were observed in the groups of maternal trees and we found no significant null alleles at any loci within any population. No significant linkage disequilibrium was observed between pairs of loci scored in maternal trees after adjustment for multiple testing.

**Table 1:** Genetic variability of *Eucalyptus gracilis* populations at eight microsatellite markers, progeny array size and seedling establishment data.

Group and parameter	Monarto Woodland	Yookamurra Sanctuary	Scotia Sanctuary
*Adults*			
* n*	20	20	18
* AR*	5.31 (0.29)	4.96 (0.31)	4.95 (0.23)
* H*_E_	0.85 (0.04)	0.81 (0.05)	0.83 (0.04)
* H*_O_	0.85 (0.04)	0.86 (0.03)	0.83 (0.03)
* F*	−0.03 (0.05)	−0.11 (0.05)	−0.04 (0.04)
*Progeny*			
Progeny array size	14.70 (0.13)	13.44 (0.20)	14.75 (0.14)
* n _planted seedlings_*	294	295	264
* n*_alive seedlings_	244	255	210
* n*_dead seedlings_	50	40	54
Establishment rate (%)	85.02	87.63	80.77
* AR*	5.17 (0.08)	4.89 (0.08)	4.92 (0.07)
* H*_E_	0.83 (0.05)	0.80 (0.05)	0.82 (0.04)
* H*_O_	0.71 (0.08)	0.72 (0.09)	0.70 (0.07)
* F*	0.17 (0.08)	0.14 (0.09)	0.16 (0.06)

*n*, number of samples.

*AR*, rarefied allelic richness.

*H*_E_ and *H*_O_, unbiased expected and observed heterozygosity, respectively.

*F*, fixation index.

standard deviations in parentheses.

### Genetic Diversity and Population Differentiation

There were no significant differences in allelic richness, expected and observed heterozygosity between progeny and maternal trees (all *t*-test *P*>0.05; Table 1). Genetic differentiation between populations was weak but significant (all genetic differentiation values <0.15; all *P*<0.05; Table S2). Yookamurra Sanctuary was more genetically similar to Monarto Woodland than Scotia Sanctuary, reflecting the spatial proximity of populations (Figure 1). Accordingly, Monarto Woodland and Scotia Sanctuary were the most genetically differentiated population pair.

### Mating System Parameters, Stand Density and Seedling Establishment

Each population was strongly outcrossed (*t*_m_ >0.95; Table 2). Biparental inbreeding and correlated paternity were generally low across populations (*t*_m_–*t*_s_ <0.20; *r*_p_<0.15), and significantly lower in Yookamurra Sanctuary than Monarto Woodland and Scotia Sanctuary. No significant differences were present in the number of full-sib groups scaled to progeny array size across populations (*k_n_* = 0.40 to 0.44). There were only weak correlations among mating system parameters when estimated at the family level (*r* ^2^<0.10), except for between correlated paternity and number of full-sibships scaled to progeny array size (*r* ^2^<0.32), the two measures of multiple paternities.

**Table 2:** Mating system parameter estimates for *Eucalyptus gracilis* from each population.

Source population	Density (trees ha^−1^)	*t*_m_	*t*_m_–*t*_s_	*r*_p_	*k_n_*
Monarto Woodland	23.67 (2.29)^a^	0.97 (0.02)^a^	0.15 (0.01)^a^	0.12 (0.02)^a^	0.40 (0.03)^a^
Yookamurra Sanctuary	49.33 (4.63)^b^	0.98 (0.01)^a^	0.11 (0.02)^b^	0.06 (0.01)^b^	0.44 (0.02)^a^
Scotia Sanctuary	20.42 (3.24)^a^	0.95 (0.02)^b^	0.16 (0.03)^a^	0.11 (0.04)^a^	0.43 (0.03)^a^

*t*_m_, outcrossing rate.

*t*_m_–*t*_s_, biparental inbreeding.

*r*_p_, correlated paternity.

*k_n_*, the number of full-sibships within progeny arrays scaled to progeny array size.

standard deviations in parentheses.

95% confidence interval homogeneous subgroups indicated by ^‘a’^ and ^‘b’^.

Seedling establishment was significantly higher at Monarto Woodland and Yookamurra Sanctuary sites than Scotia Sanctuary. There were no significant differences in seedling establishment according to seed provenance and there was no significant interaction between seed provenance and planting site (Generalized linear model: link function = binomial; seed provenance *χ* ^2^ = 2.24, *d.f.* = 2, *P* = 0.33; planting site *χ* ^2^ = 48.98, *d.f.* = 2, *P*<0.001; seed provenance × planting site *χ* ^2^ = 1.95, *d.f*. = 4, *P = *0.75; Table 1; Table S3; Table S4).

Across all families (*n* = 58), the number of full-sibships within progeny arrays (*k_n_*) and correlated paternity (*r*_p_) had strong effects on seedling establishment rate (*k_n_* had a positive effect on establishment rate: per cent deviance explained = 16.4%; *r*_p_ had a negative effect on establishment rate: per cent deviance explained = 10.3%; ΔAIC_c_ between these top two models = 3.81; ΔAIC_c_ to next best model = 5.49; ΔAIC_c_ to null model = 7.50; Table 3).

**Table 3:** General linear model comparisons of relationships between genetic predictors and establishment rate (%) of *Eucalyptus gracilis* progeny arrays.

Model	% DE	*w*AIC	ΔAIC*_c_*	*k*	*ß*
Establishmentrate ∼ *k_n_*	16.37	0.80	0.00	2	−4.09 (−9.74 to 1.52)
Establishmentrate ∼ *r*_p_	10.26	0.12	3.81	2	30.24 (−41.85 to 100.00)
Establishmentrate ∼ *t*_m_–*t*_s_	7.42	0.05	5.49	2	
Establishmentrate ∼ 1	0.00	0.02	7.50	1	
Establishmentrate ∼ *t*_m_	0.01	0.01	9.65	2	

% DE, per cent deviance explained by model.

*w*AIC, Akaike weight that shows the relative likelihood of model *i.*

ΔAIC*_c_*, indicator of differences between model AIC*_c_* (a measure of model goodness-of-fit scaled to the number of parameters in the model) and minimum AIC*_c_* in the model set.

*k*, number of parameters in each model.

*ß*, unstandardized regression slope with 5 and 95% bootstrapped percentiles in parentheses in models that were either the best fitting model or had ΔAIC*_c_* <4.

*t*_m_, outcrossing rate.

*t*_m_–*t*_s_, biparental inbreeding.

*r*_p_, correlated paternity.

*k_n_*, the number of full-sibships within progeny arrays scaled to progeny array size.

1, null model.

Biparental inbreeding had a negative effect on establishment rate, but its effect was much weaker than the number of full-sibships within progeny arrays and correlated paternity (*t*_m_–*t*_s_: per cent deviance explained = 9.07%; ΔAIC_c_ to best fitting model = 5.17). Outcrossing rate did not associate with growth (per cent deviance explained <1%; ΔAIC_c_ to best model = 9.65; ranked worse than null model).

We explored the variance of estimated family-level mating system parameters further because of possible problems of estimating these parameters from a limited progeny array sample size. We observed that most of the upper 50% of estimated mating system parameters had 95% confidence intervals that did not overlap zero, indicating significant levels of these parameters in these families, which also suggests that estimation of these parameters in our study was largely robust to our sample sizes (Figure S1). However, we do recommend that attention should be paid to the potential for high variance of family-level estimates in future studies.

**Figure 2:**
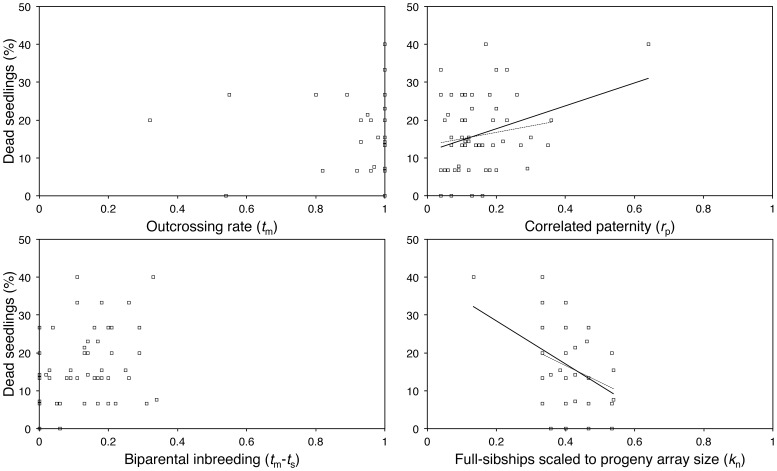
Scatterplots showing relationships between *Eucalyptus gracilis* family-level establishment rates and mating system parameters. Establishment rate percentages per progeny array are shown on the y-axis and mating system parameter values shown on the x-axis. Linear trend lines between genetic parameters and growth shown for relationships where ΔAIC*_c_* <4 (ΔAIC*_c_* values presented in Table 3). Trend lines are for all data are solid and trend lines are for data without the outlier are dashed.

We also explored the leverage and influence of the outlier on the significant regressions (see Figure 2). When the outlier was removed and these regressions were re-run, correlated paternity and the number of full-sibships within progeny arrays were still the best fitting predictors of establishment rate (*r*_p_ and *k_n_* per cent deviance explained = 16.6 and 9.3%; Table S5). Additionally, when the original regressions that included the outlier were bootstrapped, the 5 and 95% bootstrapped percentiles of the multiple paternity-establishment rate regression slopes only marginally overlapped zero (Table 2). Thus, we conclude that this outlier had high leverage but had marginal influence on the regressions and was thus retained in our analyses.

The stand density of Yookamurra Sanctuary was significantly higher than both Monarto Woodland and Scotia Sanctuary (Table 1, 2). Progeny arrays collected from Yookamurra Sanctuary exhibited significantly lower biparental inbreeding and more multiple paternities than both other populations (Table 2), and fits with expectations based on density differences between these populations. Establishment rates also tended to be higher in families from the higher density Yookamurra Sanctuary, but this effect was not significant (see text above; Table S4).

During the sampling period, rainfall was substantially higher than the long-term average at all sites (1.7, 2,1 and 2.7 times the recent past for Monarto Woodland, Yookamurra Sanctuary and Scotia Sanctuary, respectively; Table S6). This suggests that the degree of water stress acting on seedlings was somewhat lower than expected. Since selection against low fitness phenotypes should be weaker during these periods of reduced stress [60], and because it is likely that *E. gracilis* is sensitive to water availability [61], we expect to observe lower seedling mortality than during an average year. Thus, the correlations we derive between mating system parameters and fitness are probably underestimates.

## Discussion

We explored whether inbreeding avoidance in monoecious trees constrains inbreeding-fitness correlations at life stages beyond seed development [18], and whether within such systems and life stages, levels of multiple paternities had a greater influence on offspring fitness than inbreeding - the constrained inbreeding hypothesis. Further to maximising fitness in inbreeding-constrained systems, multiple paternities are likely to be positively correlated with the degree of mate discrimination/bet hedging and genetic diversity of offspring, and therefore competition among male gametes and/or female choice for superior male gametes [8], [13]. Indeed, in this study we provide evidence to support the constrained inbreeding hypothesis by showing that open-pollinated families of *Eucalyptus gracilis* had little variation in inbreeding (measuring both selfing and biparental inbreeding at the seedling stage), but demonstrated a correlation between the number of multiple paternities and fitness at the seedling life stage.

Levels of multiple paternities in *E. gracilis* seedling families should not be constrained by strong inbreeding avoidance. Therefore, as we predicted, we found that levels of multiple paternities were the strongest predictors of seedling fitness as measured by establishment rates; indeed these effects were over and above the realised effects of inbreeding-related parameters on seedlings (Table 3). Our results are consistent with previous studies that have documented fitness impacts of reduced multiple paternities, of which most of these studies were done in fragmented tree populations [11], [16], [17], [31], [32], [34].

To make our conclusion, we explored the correlations of four different mating system parameters, estimated from early seedlings for three natural populations of *E. gracilis*, with the fitness of these seedlings measured by monitoring seedling establishment rates. The four different mating system parameters we observed were two commonly used measures of multiple paternities (correlated paternity and the number of full-sibships) and two measures of inbreeding (outcrossing rate and biparental inbreeding). The two multiple paternity measures are estimated independently of inbreeding [11], [58]. Including these multiple paternity measures in our study was particularly important since species that undergo strong inbreeding avoidance when measured at the seedling life stage, like many eucalypts [3], are unlikely to express significant levels of inbreeding depression when observed at this life stage ([16], [18], but see [15]). Consequently, we suggest that in species with strong inbreeding avoidance, the degree of multiple paternities could be as important to observe as inbreeding levels when investigating intermediate stage fitness consequences of variation in mating system parameters [16]. However, with our design, we can only conclude that this fitness effect is acting on seedling establishment and not earlier (*e.g.* germination) or later life stages (*e.g*. fecundity). Thus, we encourage future studies to explore these multiple paternity effects in natural populations outside of this life stage, with special attention made to maximising the numbers of progeny used per family (to improve mating system parameter estimates), and the number of families used, since our sample size (*n* = 58 families) is low for studies of plant fitness.

It should be noted that stand density might be an important factor underlying much of the variation in observed multiple paternities here. Yookamurra Sanctuary had a stand density significantly higher than both Monarto Woodland and Scotia Sanctuary, which were similar, and accordingly Yookamurra had significantly lower biparental inbreeding and more multiple paternities. However, despite the fact that establishment rates of seedlings from Yookamurra Sanctuary tended to be higher, this effect was not significant. Thus, further investigations are required to identify the effect of population-level ecological characteristics, such as stand density, that may explain our observed patterns. However, consistent with previous studies, our data do generally support positive density-dependent establishment as a function of multiple paternity in mostly outcrossing animal-pollinated trees [16], [25].

## Supporting Information

Figure S1 **Frequency histograms of family-level estimated mating system parameters and 95% confidence intervals.**
(DOCX)

Table S1 **Genetic variability at each microsatellite locus for** ***Eucalyptus gracilis*** **maternal trees.**
(DOCX)

Table S2 **Genetic differentiation of** ***Eucalyptus gracilis*** **populations.**
(DOCX)

Table S3 **Planting sample sizes and seedling establishment information after 16 months of growth at each of the three sites and for each seed provenance.**
(DOCX)

Table S4 **Generalized linear models of effects of seed provenance and planting site on establishment rate of** ***Eucalyptus gracilis.***
(DOCX)

Table S5 **General linear model comparisons of relationships between genetic predictors and establishment rate of** ***Eucalyptus gracilis*** **without the outlier.**
(DOCX)

Table S6 **Rainfall observations collected from the closest weather stations to the planting sites with data extending >100 years.**
(DOCX)
