# Supplementary material for: Higher Levels of Multiple Paternities Increase Seedling Survival in the Long-Lived Tree Eucalyptus gracilis
Source: PLoS One. 2014 Feb 28;9(2):e90478. doi: 10.1371/journal.pone.0090478 (PMC3938745; doi:10.1371/journal.pone.0090478)
Supplement: Figure S1 — Frequency histograms of family-level estimated mating system parameters and 95% confidence intervals. (DOCX) [file pone.0090478.s001.docx]

**Figure S1**. Frequency histograms of family-level estimated mating system parameters and 95% confidence intervals.
