# Supplementary material for: Higher Levels of Multiple Paternities Increase Seedling Survival in the Long-Lived Tree Eucalyptus gracilis
Source: PLoS One. 2014 Feb 28;9(2):e90478. doi: 10.1371/journal.pone.0090478 (PMC3938745; doi:10.1371/journal.pone.0090478)
Supplement: Table S1 — Genetic variability at each microsatellite locus for Eucalyptus gracilis maternal trees. (DOCX) [file pone.0090478.s002.docx]

**Table S1**. Genetic variability at each microsatellite locus for *Eucalyptus gracilis* maternal trees (*A*, number of alleles*; H*_E_ and *H*_O_, unbiased expected and observed heterozygosity, respectively; *F*, fixation index; *Q*, probability of paternity exclusion; *QC*, combined probability of paternity exclusion; standard errors in parentheses).

| Locus | *A* | *H*_E_ | *H*_O_ | *F* | *Q* |
| --- | --- | --- | --- | --- | --- |
| Monarto Woodland |  |  |  |  |  |
| EMBRA1382 | 12 | 0.89 | 1.00 | -0.15 | 0.90 |
| EMBRA2002 | 8 | 0.88 | 0.82 | 0.04 | 0.87 |
| EMBRA1445 | 13 | 0.92 | 0.94 | -0.05 | 0.93 |
| EMBRA1284 | 12 | 0.91 | 0.82 | 0.07 | 0.92 |
| EMBRA1928 | 15 | 0.93 | 1.00 | -0.11 | 0.94 |
| EMBRA1468 | 14 | 0.94 | 0.94 | -0.04 | 0.94 |
| EMBRA1363 | 5 | 0.63 | 0.76 | -0.24 | 0.51 |
| EMBRA1363 | 5 | 0.70 | 0.65 | 0.05 | 0.62 |
| Mean | 10.50 (1.40) | 0.85 (0.04) | 0.87 (0.04) | -0.06 (0.04) | *QC* = 1.00 |
| Yookamurra Sanctuary |  |  |  |  |  |
| EMBRA1382 | 12 | 0.88 | 0.85 | 0.01 | 0.88 |
| EMBRA2002 | 10 | 0.85 | 0.95 | -0.14 | 0.86 |
| EMBRA1445 | 10 | 0.75 | 0.85 | -0.16 | 0.74 |
| EMBRA1284 | 14 | 0.91 | 0.90 | -0.01 | 0.92 |
| EMBRA1928 | 15 | 0.93 | 0.95 | -0.04 | 0.95 |
| EMBRA1468 | 16 | 0.92 | 0.90 | 0.00 | 0.94 |
| EMBRA1363 | 4 | 0.54 | 0.75 | -0.41 | 0.40 |
| EMBRA1363 | 5 | 0.70 | 0.75 | -0.09 | 0.63 |
| Mean | 10.75 (1.57) | 0.81 (0.04) | 0.86 (0.03) | -0.11 (0.05) | *QC* = 1.00 |
| Scotia Sanctuary |  |  |  |  |  |
| EMBRA1382 | 10 | 0.87 | 0.83 | 0.01 | 0.86 |
| EMBRA2002 | 9 | 0.87 | 0.94 | -0.12 | 0.86 |
| EMBRA1445 | 10 | 0.80 | 0.72 | 0.07 | 0.80 |
| EMBRA1284 | 11 | 0.90 | 0.83 | 0.05 | 0.90 |
| EMBRA1928 | 13 | 0.90 | 0.94 | -0.08 | 0.91 |
| EMBRA1468 | 11 | 0.88 | 0.83 | 0.03 | 0.89 |
| EMBRA1363 | 5 | 0.58 | 0.72 | -0.29 | 0.46 |
| EMBRA1363 | 8 | 0.80 | 0.78 | 0.00 | 0.77 |
| Mean | 9.63 (0.84) | 0.83 (0.04) | 0.83 (0.03) | -0.04 (0.04) | *QC* = 1.00 |
