# Supplementary material for: Higher Levels of Multiple Paternities Increase Seedling Survival in the Long-Lived Tree Eucalyptus gracilis
Source: PLoS One. 2014 Feb 28;9(2):e90478. doi: 10.1371/journal.pone.0090478 (PMC3938745; doi:10.1371/journal.pone.0090478)
Supplement: Table S2 — Genetic differentiation of Eucalyptus gracilis populations. (DOCX) [file pone.0090478.s003.docx]

**Table S2**. Genetic differentiation of *Eucalyptus gracilis* populations (*G*_ST_est_ above the diagonal and *D*_est_ below; *P* < 0.05, < 0.01 indicated by *, **).

| Source population | Monarto Woodland | Yookamurra Sanctuary | Scotia Sanctuary |
| --- | --- | --- | --- |
| Monarto Woodland | - | 0.05* | 0.13** |
| Yookamurra Sanctuary | 0.04* | - | 0.08** |
| Scotia Sanctuary | 0.11** | 0.06** | - |
