# Supplementary material for: Higher Levels of Multiple Paternities Increase Seedling Survival in the Long-Lived Tree Eucalyptus gracilis
Source: PLoS One. 2014 Feb 28;9(2):e90478. doi: 10.1371/journal.pone.0090478 (PMC3938745; doi:10.1371/journal.pone.0090478)
Supplement: Table S3 — Planting sample sizes and seedling establishment information after 16 months of growth at each of the three sites and for each seed provenance. (DOCX) [file pone.0090478.s004.docx]

**Table S3**. Planting sample sizes and seedling establishment information after 16 months of growth at each of the three sites and for each seed provenance.

| Site | *n* _planted_ | *n* _alive_ | *n* _dead_ |
| --- | --- | --- | --- |
| *Monarto Woodland site* | 287 | 264 | 23 |
| Monarto Woodland source | 99 | 93 | 6 |
| Yookamurra Sanctuary source | 99 | 91 | 8 |
| Scotia Sanctuary source | 89 | 79 | 10 |
| *Yookamurra* *Sanctuary site* | 277 | 244 | 33 |
| Monarto Woodland source | 96 | 83 | 13 |
| Yookamurra Sanctuary source | 97 | 88 | 9 |
| Scotia Sanctuary source | 84 | 72 | 12 |
| *Scotia Sanctuary site* | 289 | 205 | 84 |
| Monarto Woodland source | 99 | 68 | 31 |
| Yookamurra Sanctuary source | 99 | 76 | 23 |
| Scotia Sanctuary source | 91 | 59 | 32 |
