# Supplementary material for: Higher Levels of Multiple Paternities Increase Seedling Survival in the Long-Lived Tree Eucalyptus gracilis
Source: PLoS One. 2014 Feb 28;9(2):e90478. doi: 10.1371/journal.pone.0090478 (PMC3938745; doi:10.1371/journal.pone.0090478)
Supplement: Table S4 — Generalized linear models of effects of seed provenance and planting site on establishment rate of Eucalyptus gracilis. (DOCX) [file pone.0090478.s005.docx]

**Table S4**. Generalized linear models of effects of seed provenance and planting site on establishment rate of *Eucalyptus gracilis* (*n* _seedlings_ = 853; link function = binomial; response variable = seedling survival).

| Parameter | Likelihood ratio *χ*^2^ | *d.f.* | *P* |
| --- | --- | --- | --- |
| Intercept | 441.71 | 1 | <0.001 |
| Seed provenance | 2.24 | 2 | 0.326 |
| Planting site | 48.98 | 2 | <0.001 |
| Seed provenance x planting site | 1.95 | 4 | 0.745 |
