# Supplementary material for: Higher Levels of Multiple Paternities Increase Seedling Survival in the Long-Lived Tree Eucalyptus gracilis
Source: PLoS One. 2014 Feb 28;9(2):e90478. doi: 10.1371/journal.pone.0090478 (PMC3938745; doi:10.1371/journal.pone.0090478)
Supplement: Table S5 — General linear model comparisons of relationships between genetic predictors and establishment rate of Eucalyptus gracilis without the outlier. (DOCX) [file pone.0090478.s006.docx]

**Table S5**. General linear model comparisons of relationships between genetic predictors and establishment rate (%) of *Eucalyptus gracilis* families without the outlier (% DE, per cent deviance explained by model; *w*AIC, Akaike weight that shows the relative likelihood of model *i*; ΔAIC*_c_*, indicator of differences between model AIC*_c_* and minimum AIC*_c_* in the model set; *k*, number of parameters in each model; *t*_m_, outcrossing rate; *t*_m_-*t*_s_, biparental inbreeding; *r*_p_, correlated paternity; *k_n_*, the number of full-sibships within progeny arrays scaled to progeny array size; 1, null model).

| Model | % DE | *w*AIC | ΔAIC*_c_* | *k* |
| --- | --- | --- | --- | --- |
| Establishment rate ~ *r*_p_ | 16.60 | 0.85 | 0.00 | 2 |
| Establishment rate ~ *k_n_* | 9.32 | 0.09 | 4.44 | 2 |
| Establishment rate ~ *t*_m_-*t*_s_ | 6.03 | 0.04 | 6.33 | 2 |
| Establishment rate ~ 1 | 0.00 | 0.02 | 7.46 | 1 |
| Establishment rate ~ *t*_m_ | 0.17 | 0.01 | 9.53 | 2 |
