# Supplementary material for: Higher Levels of Multiple Paternities Increase Seedling Survival in the Long-Lived Tree Eucalyptus gracilis
Source: PLoS One. 2014 Feb 28;9(2):e90478. doi: 10.1371/journal.pone.0090478 (PMC3938745; doi:10.1371/journal.pone.0090478)
Supplement: Table S6 — Rainfall observations collected from the closest weather stations to the planting sites with data extending >100 years. (DOCX) [file pone.0090478.s007.docx]

**Table S6**. Rainfall observations collected from the closest weather stations to the planting sites with data extending >100 years (data sourced from Australian Bureau of Meteorology; www.bom.gov.au).

| Planting site | Collection date | Rainfall (mm) ± SE |
| --- | --- | --- |
| Monarto Woodland (Callington station) | 1883-2009 | 377 ± 8 |
|  | May 2010-May 2011 | 665 |
| Yookamurra Sanctuary (Sedan station) | 1881-2009 | 294 ± 10 |
|  | May 2010-May 2011 | 623 |
| Scotia Sanctuary (Wentworth station) | 1868-2009 | 284 ± 8 |
|  | May 2010-May 2011 | 774 |
